# Determination of Transport Properties of Glycol-Based NanoFluids Derived from Surface Functionalized Graphene

**DOI:** 10.3390/nano9020252

**Published:** 2019-02-12

**Authors:** Ebtisam Saeed, Manuel M. Piñeiro, Carolina Hermida-Merino, María José Pastoriza-Gallego

**Affiliations:** 1 Chemistry and Physics Department, Faculty of Science, Beni-Suef University, Beni Suef 62511, Egypt; ebtsamtarek@psas.bsu.edu.eg; 2 Departamento de Física Aplicada, Facultad de Ciencias, Universidade de Vigo, 36310 Vigo, Spain; cahermida@uvigo.es (C.H.-M.); mjpg@uvigo.es (M.J.P.-G.)

**Keywords:** propylene glycol, graphene, nanofluids, viscoelasticity, thermal conductivity

## Abstract

Suspensions of nanometric-sized graphene platelets have been proposed recently as potential heat exchange working fluids, due to their remarkably enhanced thermal profile. Nevertheless, their use presents serious long-term stability issues. Due to this limitation, the nanoplatelets surface chemical functionalization has been postulated as a promising alternative to solve this problem. In this work, graphene nanoplatelets were functionalized following an oxidation-reduction process, and then dispersed in glycol as base fluid. The nanoparticles chemical profile was determined using XPS (x-ray photoelectron spectroscopy). The thermo-physical properties characterization of these nanofluids was performed by determining their viscosity and thermal conductivity, because of their impact on practical applications related with fluid flow and heat transfer. The effect of temperature and shearing time on viscosity were analyzed. Viscosity was measured with a stress-controlled rheometer. All samples show shear-thinning behavior with a very remarkable influence of temperature in their viscoelastic profile.

## 1. Introduction

Concerns about the future of energy supply availability, together with efficiency issues, or global change evidence are some of the main reasons supporting the huge efforts performed in the optimization of energy production processes, and on consumption rationalization. The required improvements in this area need a great deal of innovation, and a particularly concerned sector is that of cooling and thermal management in industrial processes involving heat transfer processes.

The optimization of the heat transfer efficiency of conventional fluids is necessary to improve the heat dissipation, energy efficiency, and the device lifetimes. This target entails bringing improvement in diverse thermo-physical properties affecting working fluid flow and heat transfer including thermal conductivity, viscosity, density, and specific heat can be pointed out for their influence [1,2].

A revision of literature reveals that rigorous theoretical and experimental studies were recently devoted to increase the thermal conductivity of liquids by using the suspension of small particles. Nevertheless, the limited stability of the resulting suspensions is a key concern for micrometric-sized colloids. The use of dispersed nanometric size nanoparticles has been shown to largely overcome this stability issue. Due to the high surface-to-volume ratio of the nanoparticles, they remain in suspension for long periods. They are also suitable for their use in microsystems since they avoid channel clogging in applications as microelectronics heat dissipation.

The application of nanotechnology in this scenario of thermal engineering applications, in the earlier 1990s when the concept “nanofluid” became popular and widely used, denotes solid nanometric-sized colloidal suspensions in different base fluids. These nanoparticles, which may be of a different chemical nature, range from pure metals, metallic oxides, alloys, semiconductors, and different carbon allotropes as nanotubes or graphene derivatives. The nanoparticles are suspended by the combination of several effects, as Brownian motion and electrostatic repulsion between the double charged layers in their surface. When they are in equilibrium with no flow, they are distributed in a balance between buoyant weight and thermal agitation. When these nanocolloids were first introduced showing the first experimental evidences of non-trivial enhanced heat transfer properties and transport properties, the number of related studies in scientific literature has grown almost exponentially [3,4,5,6]. The initial declared fabulous enhancements in heat transfer ability were later tuned down, and some principles were agreed on regarding the need to establish rigorous experimental standards to ensure sample reproducibility and data reliability. After a rush period, these systems are being gradually understood even though many questions remain unanswered.

Fluid viscosity undoubtedly represents a relevant variable in heat transfer and flow applications. In this paper, the rheological behavior of several nanofluid samples will be investigated. This behavior is affected by the nanofluid preparation method, the nanoparticles size and shape, concentration, viscosity of the base fluid, temperature, potential surfactant use, and dispersion state of the suspended material.

Since the earliest stage of their introduction, the enhanced heat transfer properties of nanofluids have become a very active research area, and the number of related scientific publications still continue showing an impressive growth. Despite this large research body, most of the publications have focused on a rather narrow target and some crucial topics, such as nanofluid rheological behavior, has been scarcely addressed. This fact is somewhat puzzling, since a precise determination of this type of properties is a key to determine their suitability for most heat transfer applications [7]. Actually, mass transport properties, and viscoelastic behavior, are as relevant as thermal transport properties in the technical profile of any potential working fluid in these applications. The pumping power required for a given fluid is dependent on the pressure drop, which is dependent on the fluid viscous behavior. The ideal profile for a working fluid in a thermal engineering application should combine ideally simultaneous low viscosity and high thermal conductivity. A small improvement in heat transfer leads to a large impact on the design requirement of the heat flow application with evident economy efficiency implications. For the case of solid colloidal dispersions as nanofluids, the dispersion and stability conditions have to be considered carefully as key issues that must be addressed when planning any industrial application [8].

In a recent study [9], we have analyzed the properties of exfoliated graphene nanosheets (xGnP) derived nanofluids, and the effect of chemical surface functionalization in their thermophysical profile. The comparison of the viscosity trends showed that the oxidation process on these nanosheets produced a remarkable viscosity increase, which turned out to be shear dependent and more evident in the low shear region. The results of the deformation sweep tests revealed that the exfoliated graphene oxide nanosheets (xGOnP) structural interactions are considerably stronger than in the case of the non-oxidized version. This is an effect of the increase of surface bonded oxygen-containing functional groups because of the oxidation process. At the same time, the surface adsorbed oxygen decreases nanosheets hydrophobicity, which leads to a solubility increase and produces a complexified aggregate structure as final effect. This evolution in the nanofluid internal structure, which is shown as a reflection on the non-Newtonian rheological profile, was highlighted because then chemical surface functionalization arose as a potential tool to be used for smart nanofluid design and synthesis tailoring for different heat transfer processes. The analysis of thermo-physical profiles of nanofluids derived from different carbon allotropes is an active research area, which is shown by the large number of recent studies presented [10,11,12].

The objective of the present work is to go forward in characterizing this innovative category of graphene derived nanofluids, which, in this case, present the study of the results of surface functionalization on thermal conductivity and a rheological profile of graphene oxide nanosheets dispersed in propylene glycol as the base fluid (in the following, denoted as PG-xGOnP for brevity).

## 2. Experimental

### 2.1. Materials

Exfoliated graphite nanoplatelets (xGnP-Grade H) were obtained from XG Sciences, Inc. (Lansing, MI, USA). xGnP-H individual nanoparticles are constituted by several layers of exfoliated graphite. The provider declares for this product an average surface area in the interval (50, 80) m^2^/g, with an estimated individual aggregate thickness of 15 nm and diameter around 5 μm. The base fluid used was Propylene glycol (PG), supplied by Aldrich ≥99.5%, Madrid, Spain). Other products used during the synthesis protocol were NaNO_3_ (Sigma-Aldrich, ≥99%, Madrid, Spain), H_2_SO_4_, (Sigma-Aldrich, 95%–98%, Madrid, Spain), KMnO_4_ (Sigma-Aldrich, ≥99%, Madrid, Spain), H_2_O_2_ (Sigma-Aldrich, Madrid, Spain) and N_2_H_4_·xH_2_O (Sigma-Aldrich, 50%–60%, Madrid, Spain). 

### 2.2. Synthesis

For a rigorous and reliable optimization of a given nanofluid thermal profile, the first step required is a well-defined and reproducible synthesis protocol, intended to produce homogeneous and long-term stable suspensions. The remarkably large number of potential alternatives of nanoparticle-base fluid combinations has to be emphasized when considering a nanofluid as a potential working fluid, and the final choice has to be guided by the technical requirements of the particular application of interest.

#### 2.2.1. Synthesis of Graphene Oxide (xGOnP) Nanoparticles

The first step was the preparation of graphene oxide using a modification of the Hummers and Offeman’s method [13]. A total of 2 g of graphene nanosheets (xGnP), 2g NaNO_3_, and 23mL H_2_SO_4_ (98%) (obtained by mixing and dissolving first NaNO_3_ in sulfuric acid) were used in the reaction, and then stirred in an ice bath. Then, 6g of KMnO_4_ were added at a controlled slow rate under agitation, which always kept the suspension temperature below 20 °C. The mixture was afterwards stirred in an ice bath for 2 h, and heated to 35 °C during a 30-min period.

After this period, 92 mL of 70 °C water were slowly added (1.5 mL/min). The heat produced heated the fluid up to 98 °C. The resulting brown colored suspension was kept at 98 °C for 15 min. Afterward, 280 mL of water were added at 70 °C, and the reaction was stopped with the addition of 40 mL of 30 wt %. hydrogen peroxide solution. The solid was then separated by combining centrifugation and hot water washing. The resulting xGOnP nanosheets were dried during three days at 55 °C.

#### 2.2.2. Production of Reduced Graphene Oxide (r-xGOnP) Nanoparticles

**Figure 1:**
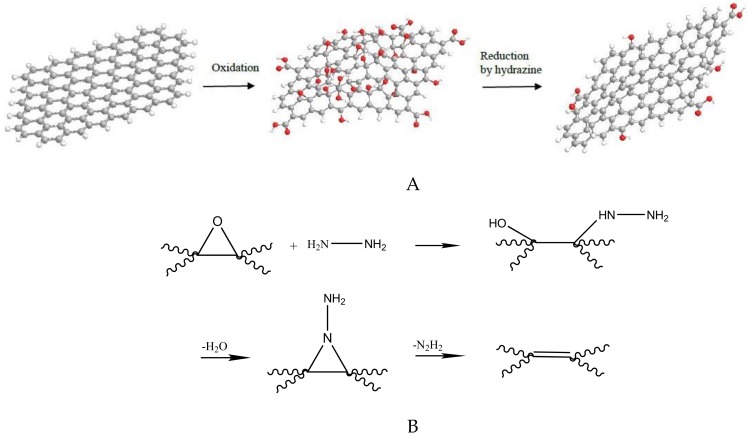
Sketch of the experimental process followed for oxidation (**A**) and posterior epoxy reduction (using hydrazine) (**B**) of graphene nanosheets [the process is described in detail in [15]].

Reduced graphene oxide was prepared via 2 g GO in a 500 mL round bottom flask, with 400 mL of de-ionized water. After continuous stirring and 30 min of sonication, 10 mL 50% hydrazine hydrate were added. After that, the mixture was maintained under stirring and heating (100 °C) in a water-cooler condenser for 24 h, where r-xGOnP precipitated as a black solid. This solid was filtrated, the water was washed (5 × 100 mL), and dried. The previous steps were repeated three times [14]. The process of nanosheets oxidation and reduction is outlined in Figure 1.

### 2.3. Characterization

With the aim to understand the modifications on the nanoplatelet original surface, a set of different characterization techniques was applied. This set included scanning electron microscopy (SEM), X-ray photoelectron spectroscopy (XPS), and Fourier transform infrared spectroscopy (FTIR). These analytical techniques are used to determine the newly created surface functional groups through elemental analysis, and characterization of crystallinity, aggregation, and structure of the analyzed nanoparticles.

Scanning electron microscopy (SEM) determined the morphology of the dry nanopowder. A field emission gun JEOL JSM-6400-SEM (JEOL, Tokyo, Japan), at an acceleration voltage of 20 kV in the electro-backscatter image was used. SEM samples were prepared by deposition of the nanopowder on a brass support with a double-sided conductive strip. This device incorporates an energy dispersive X-ray spectrometer (EDS) providing chemical characterization of the samples.

X-ray photoelectron spectroscopy (XPS) was obtained from a Thermo Scientific K-Alpha ESCA (Waltham, MA, USA), using a monochromatic radiation source Al-Kα1,2 (1486.92 eV). The photoelectrons were collected at a 90° angle from the sample surface. The constant analyzer (CAE) energy mode was used with a step power of 20 eV for the study, and 100 eV for the high-resolution spectra. The atomic percentage compositions were determined from XPS spectra, considering integrated peak areas using the Scofield sensitivity factors and the Shirley background subtraction method. The adjustment of the Corel level curve was obtained from the convolution of Gaussian and Lorentzian functions.

Concerning Fourier transform infrared spectroscopy (FT-IR), the experimental setup used was a Bruker Vector 22 spectrometer operating in a transmission mode, using potassium bromide pellets for 32 scans in the range of 400 to 4000 cm^−1^, which estimates a resolution better than 4 cm^−1^.

The stability of nanofluids obtained from original and functionalized nanoplatelets were checked by evaluating the concentration influence. Thermal conductivity and the rheological properties of the nanofluids were determined experimentally at different concentrations.

### 2.4. Sample Preparation and Stability

We have used the two-step method to produce the nanofluid samples. First, the nanoparticles are prepared, and then they are dispersed in the base fluid. This method is quite straightforward but it may lead to nanoparticle agglomeration and poor nanofluid stability. Low nanoparticles charges are then necessary to ensure proper stability without the addition of chemical surfactants.

Suspensions of xGnP, xGOnP, or r-xGOnP in propylene glycol (PG) were analyzed. An electronic balance Mettler AE-240 (Mettler Toledo, Greifensee, Switzerland), (5 × 10^−5^ g accuracy) was used to weigh the nanoparticle powder and then it was dispersed in a predetermined volume of the base fluid to obtain the desired weight fraction, which may be as high as 3% in this case. The particles were dispersed by immersing the nanofluid samples within an ultrasonic bath (Clifton, Fisher Sci., Leicestershire, UK), during 120 min at a frequency of 50 to 60 Hz and a power of 80 W, considering a density of 2.2 g·cm^3^ for the xGnP nanopowder.

With respect to the stability of nanofluids (NF), the high surface activity of nanomaterials induces aggregation. Due to the agglomeration of the nanoparticles, obstruction and settlement inside micro-channels occurs, which spoils the nanofluid utility. In previous studies, it has been shown that the most relevant factors determining the nanofluid stability are the size, shape, concentration, and chemical nature of the nanoparticle together with pH and polar/non-polar, and viscous nature of the base fluid. Lastly, the dispersion process used also plays a relevant role.

The stability of the studied nanofluids was analyzed with a Thermo Scientific Helios-Omega Ultraviolet-Visible (UV-Vis) spectrometer (Waltham, MA, USA). It is equipped with a thermo-stated cell carrier, where samples undergoing different sonication times were tested. This technique has been extensively used to analyze nanofluids for its rather simple use and fast response. Stability is determined by monitoring the absorbance time evolution of a light beam passing through the fluid sample and operating with a wavelength in the (190 to 1100) nm range. The nature of this technique restricts its application to low concentration nanofluids, since increasing concentration rapidly turns samples opaque. 

### 2.5. Thermal Conductivity

The unusually high thermal conductivity values reported initially for nanofluids were the boosting factor for the interest in this type of systems. Since then, many studies have been devoted to the analysis of this property. The transient hot wire method (THW, Kestin and Wakeham, 1978) [16] is a very common technique for thermal conductivity experimental determination, and has been widely applied to nanofluids [5,17,18]. This technique, proposed originally by Stâlhane and Pyk [19], was developed to determine absolute thermal conductivity of powders and solids. Groot et al. [20] introduced the use of this technique for homogenous fluids. The measuring probe consists of a linear wire inserted into the fluid to be measured. The wire serves two different purposes, as it is used as a heater, through a controlled current circulating to generate heat by the Joule effect, and it also acts as a thermometer. The theoretical basis is the equation introduced by Carslaw and Jaeger [21], corresponding to an ideal model of the temperature evolution of a thin infinite zero mass linear heat source, placed within an infinite medium.

(1) $$k=\frac{q\left(\ln t_{2}-\ln t_{1}\right)}{4\pi \left(\Delta T_{2}-\Delta T_{1}\right)},$$

In this equation, q represents the constant heat rate applied to the thin and infinitely long linear source, while Δ*T* is the temperature difference between the medium and the initial temperature, and Δ*T*_1_ and Δ*T*_2_ are the temperature variations at *t*_1_ and *t*_2_, respectively.

From a theoretical perspective, there is not a satisfactory and agreed theory able to describe the physical foundations of this nanofluid property, and the different competing heat transfer mechanisms are far from being well understood. Since the topic is currently quite controversial, far more rigorous experimental contributions are needed to deepen knowledge on these quite intriguing nanoscale colloids.

Thermal conductivity data presented in this paper were measured with a KD2 (Decagon Devices, Inc., Pullman, WA, USA). Arranged with a KS-1 probe of 1.3 mm diameter and 60 mm long, which can measure thermal conductivities in the range (0.02 to 2) W·m^−1^·K^−1^. Samples were initially thermo-stated in a Grant GP200 oil bath (Grant Instruments, Cambridge, UK). The estimated standard uncertainty for the thermal conductivity probe is lower than 0.010 W·m^−1^·K^−1^, for the range (0.02 to 0.2) W·m^−1^·K^−1^, and 3% for the interval (0.2 to 2) W·m^−1^·K^−1^.

### 2.6. Non-Linear Viscoelastic Measurements

In comparison with the relatively abundant number of experimental studies on the thermal conductivity of nanofluids, rheological studies are still very rare in literature. A rheometer determines the relationship between the speed of rotation of a solid (graphene) in a viscous medium (propylene glycol) and the applied force or torsion. In this study, a rheometer with a cone-plate geometry has been used. This is probably the most widely used device for the study of rheological properties of non-Newtonian fluids [17,22,23]. This geometry results in a uniform shear rate and viscosity is obtained using the formula below.

(2) $$\eta =\frac{3\;T\;\theta_{0}}{2\pi \;R^{3}\;\Omega_{0}}$$

In the latter expression, *θ*_0_ is the cone-plate angle (*θ*_0_ < (0.05–0.1) rad), *T* is the total torque, Ω_0_ is the relative angular velocity, and *R* is the cone radius.

A Physica MCR 101 rheometer (Anton Paar, Graz, Austria), with a cone-plate geometry (CP 25-1) was used in this work. It was able to control torques between 0.5 µN·m and 125 mN·m and normal force from 0.1 to 30 N. The cone-plate gap was 0.048 mm, and the in-built Peltier system was used to control the sample temperature. Non-linear viscoelastic experiments (flow curves) were performed at shear rates up to 1000s^−1^, varying the temperature at concentrations up to 3 wt % of xGnP/PG, xGOnP/PG and r-xGOnP/PG nanofluids. Three replicas for each experiment were carried out.

## 3. Results and Discussion

### 3.1. Characterization

**Figure 2:**
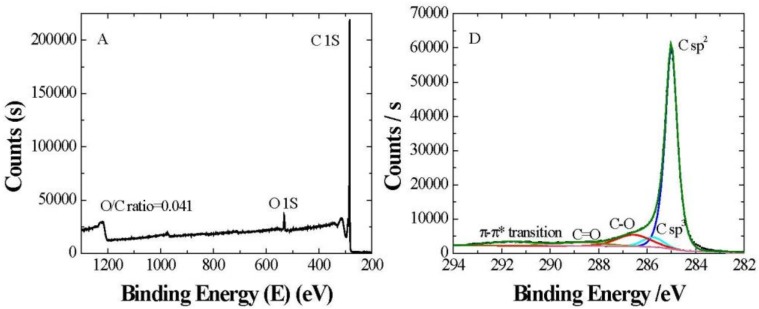

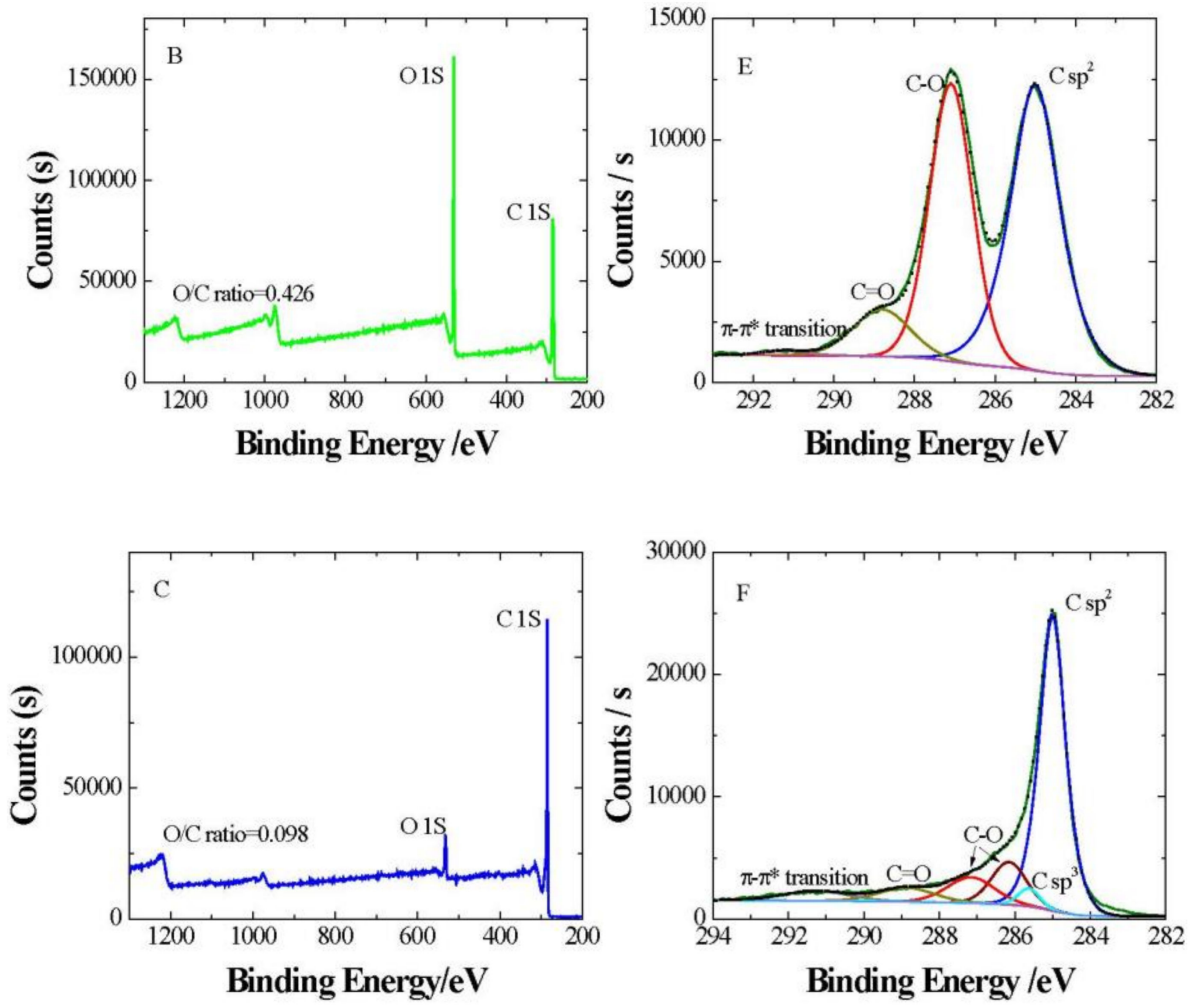
Full XPS spectra of (**A**) xGnP, (**B**) xGOnP, and (**C**) r-xGOnP nanosheets. High-resolution C1s spectra of (**D**) xGnP, (**E**) xGOnP, and (**F**) r-xGOnP nanosheets.

The XPS analytical technique allows a quantitative elemental analysis of surfaces even though its sensitivity does not allow to detect all types of functional groups [24]. This is because the relative chemical shifts of certain groups are below the available energy resolution and the intrinsic peak widths produce undesired overlaps between them [23,25]. For the case of interest of carbon atoms, they can be resolved for the number of bonds with oxygen atoms, which allows functional group identification. This means that a C–O will produce a different shift than an O–C–O or a C–C. Nevertheless, hydroxyl groups cannot be discerned from peroxides and epoxides due to their C1s binding energies [26]. Figure 2 shows the full XPS spectra of xGnP-H, xGOnP, and r-xGOnP. The relative atomic percentages of C1s peaks and O 1s peaks, with their element ratio, are shown in Table 1.

The O/C ratio of xGOnP obtained from elemental analysis of XPS spectrum is much higher than that of xGnP and r-xGOnP. It indicates that oxidation has taken place, and, during the reduction process, the oxygen groups are reduced.

The de-convoluted C1s peaks of xGnP, GONs, GONs-OA, and GONs-OA-PP, displayed in Figure 2, and their relative atomic percentages, are gathered in Table 2. The C1s XPS spectrum consists of six different chemically shifted components, which can be assigned as follows: the peak at 285eV (C1) corresponding to the graphitic structure (C=C), that at 285.6–285.7eV (C2) for sp3-hybridized carbon atoms (C–C), and those at 286.6 (C3) and 288.3–289.2 eV (C4) attributed to C-O and C=O, respectively. Lastly, the band at 290.2 eV (C5) corresponds to the π-π* transition peak [27].

As shown in Table 2, in the case of xGnP and GONs, the peak fractions of C=C, C-C, and the π-π* transition decrease, while those of –C–O and C=O are increased. This may be interpreted assuming that oxidation occurred on xGnP surfaces by the dissociation of the active π bonds in C=C bonds and the reactions with the defect carbons. After reduction, r-xGOnP, a decrease in the C-O and C=O peaks can be noted, and an increase in the C=C, C–C peaks, and the π-π* transition, which recovers part of the initial structure but with more oxygen incorporated in the nanosheets surface.

**Figure 3:**
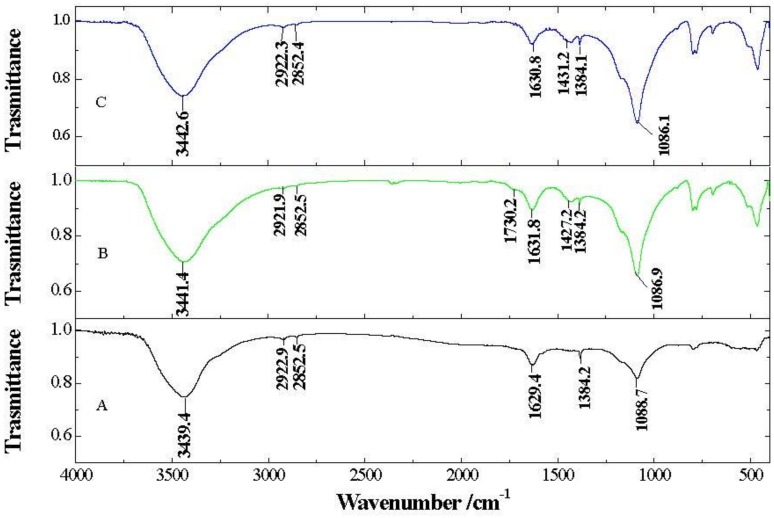
FT-IR spectra of xGnP (**A**), xGOnP (**B**), and r-xGOnP (**C**).

Figure 3 displays Fourier transform Infrared Spectroscopy (FT-IR) absorption bands of xGnP, xGOnP, and r-xGOnP including O-H stretching vibration (3440 cm^−1^) and C–O–C stretching (epoxy group) vibrations (1087 cm^−1^). The intensive band at 1384 cm^−1^ can be identified with the C–H bending vibration absorption. The asymmetric bands of the alkyl group at 2852 and 2922 cm^−1^ belong to the C-H stretching vibration. The band appearing at 1630 cm^−1^ can be attributed to the absorption of the C=C stretching aromatic groups and O–H bending [28,29].

The peak at 1087 cm^−1^, corresponding to epoxy groups, increases in the oxidation process (Figure 3B) and new bands appear at 1430 cm^−1^ and 1730 cm^−1^, for COO– symmetric stretching and C=O stretching carboxyl groups situated at the edges of graphene sheets, respectively, which indicates an increase of oxygen in the sample. In the reduction process (Figure 3C), the band at 1730 cm^−1^ disappears, and the band at 3440 cm^−1^ decreases in intensity, which indicates a reduction in the hydroxyl and carboxyl groups, and, thus, a decrease of the oxygen presence in the sample.

**Figure 4:**
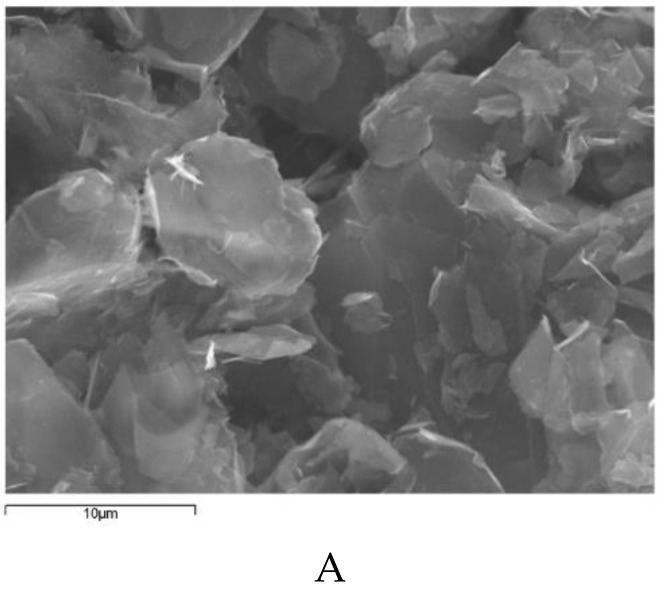

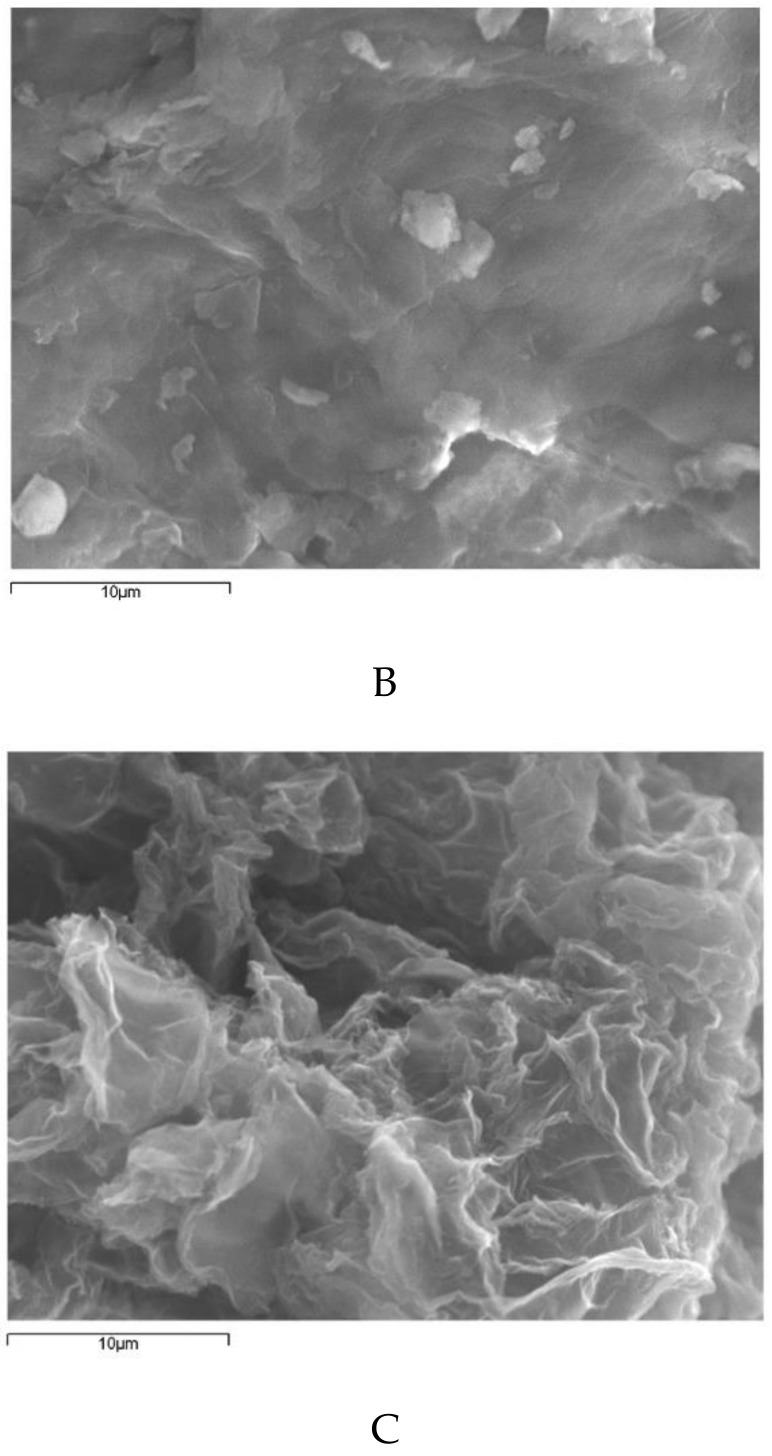
FEG-SEM, 3500×, JEOL JSM-6400 image of (**A**) xGnP-H, (**B**) xGOnP, (**C**) rxGOnP.

The geometry of the nanopowder was evaluated using Scanning Electron Microscopy (SEM). Typical SEM images of xGnP-H, xGOnP, and r-xGOnP are represented in Figure 4, which shows that the nanoplatelets are crumpled and folded. It is clear that these bidimensional are thermodynamically more stable when they are bent, which was also found to occur in grade C graphene nanoparticles (xGnP-C) [22].

Energy dispersive spectroscopy (EDS) was used to obtain the chemical composition. Table 3 shows the atomic percentage of the principal elements in the nanosheet samples. A small percent of impurities (< 1%) is revealed.

### 3.2. Stability

**Figure 5:**
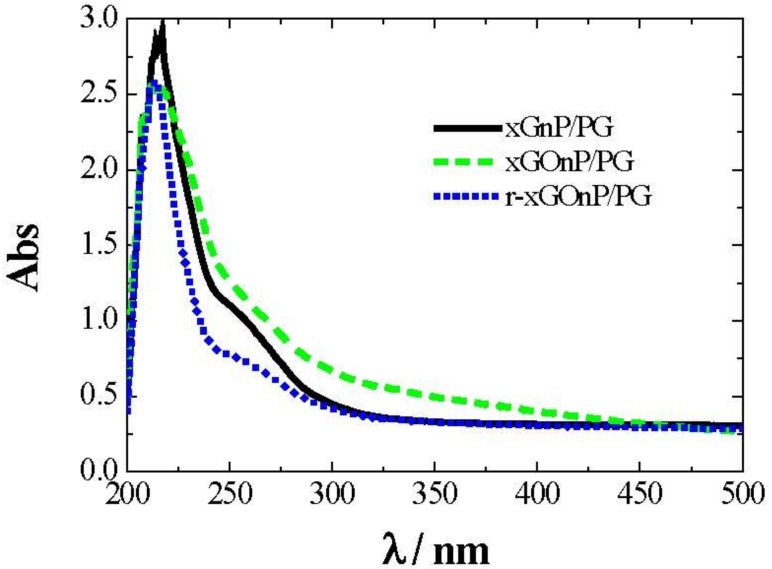
UV-Vis absorption spectra of xGnP/PG (solid line), xGOnP/PG (dashed line), and r-xGOnP/PG (dotted line) nanofluids, 0.003 wt %, *T* = 298.15 K.

The stability of xGnP/PG, xGOnP/PG, and r-xGOnP/PG was monitored at different sonication times, as described using a UV/Vis spectrometer. Figure 5 shows optical absorption spectra for a nanofluid sample, considering at 0.003 wt. % so that the absorbance value does not exceed the detection limits of the equipment.

**Figure 6:**
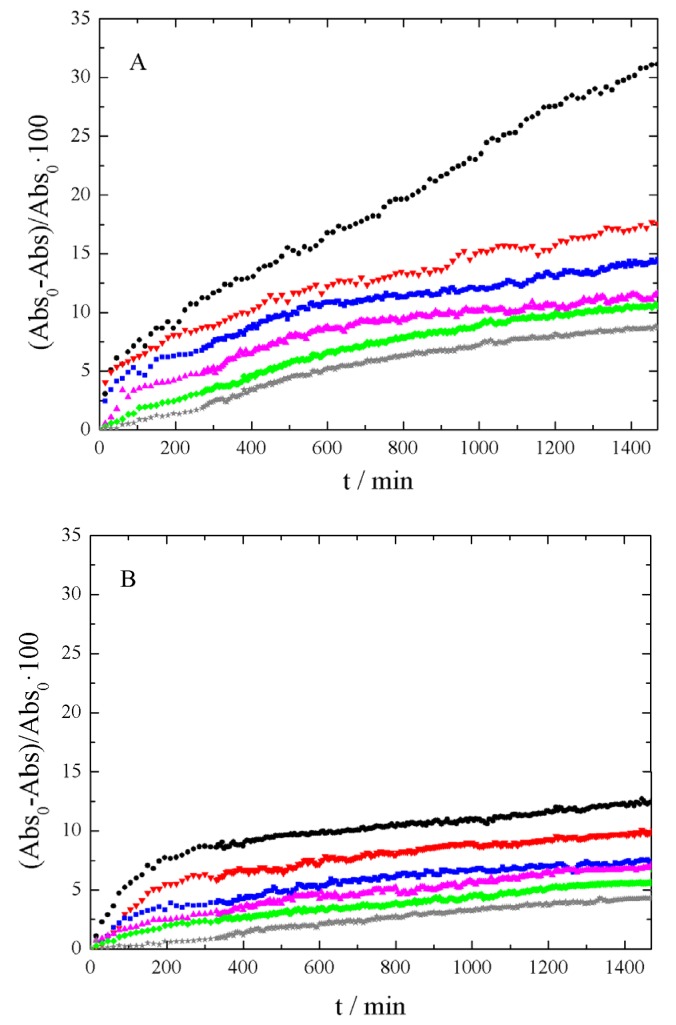

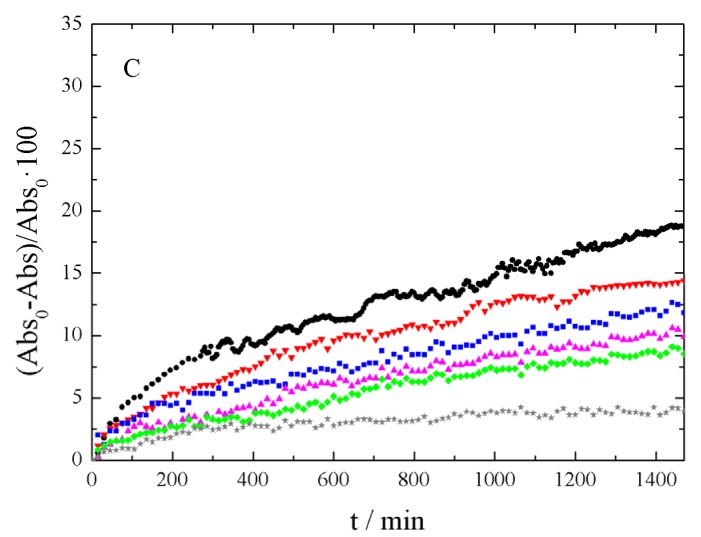
Absorbance decrease, 100 (Abs0 - Abs)/Abs0, at λ = 250 nm and different sonication time (● 1 h, ▼2 h, ■3 h, ▲4 h, ♦5 h, and ★6 h) of (**A**) xGnP/PG, (**B**) xGOnP/PG and (**C**) r-xGOnP/PG nanofluids, 0.003 wt%, T = 298.15 K.

After evaluating the results of Figure 5, the wavelength was then fixed at λ = 250 nm. With this setting, the absorbance time evolution was analyzed, depending on the sonication time employed for each sample. Figure 6 shows the absorbance decrease recorded for xGnP/PG, xGOnP/PG, and r-xGOnP/ PG nanofluids. Optimal stability is obtained in all cases, for the time range studied, after 6 h of sonication. After 24 h, the absorbance xGnP/ PG decreases less than 9%, and less than 4% for xGOnP/PG and r-xGOnP/PG. The stability is better for xGOnP/PG nanofluids. After the chemical reduction process, the stability decreases for the reduction of hydroxyl groups in the surface of graphene nanosheets. In spite of this, the stability of r-xGOnP/PG can be regarded as good enough, and it is better than that of xGnP/PG.

### 3.3. Thermal Conductivity

**Figure 7:**
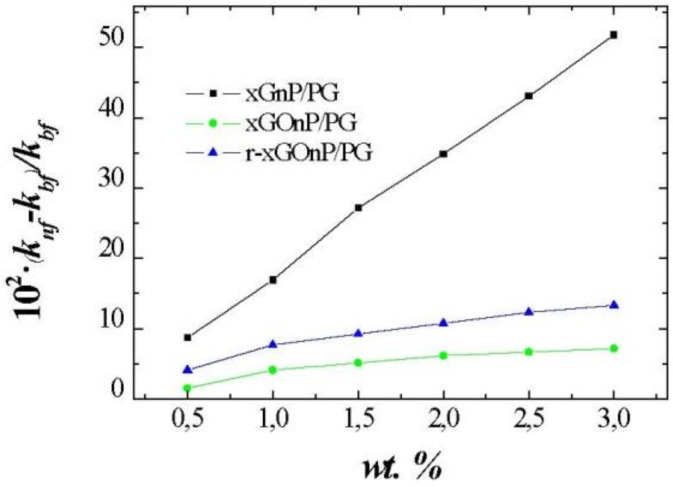
Thermal conductivity enhancements, 100(k_nf_-k_bf_)/k_bf_, for different weight fractions, wt. %, of xGnP/PG, xGOnP/PG, and r-xGOnP/PG nanofluids at T = 323.15 K.

Experimental thermal conductivities for xGnP, xGOnP, and rxGOnP / PG NFs, and also for the plain base fluid are presented in Table 4. For the base fluid, maximum absolute deviations between literature data and our experimental values are lower than 0.003 W·m^−1^·K^−1^, [30,31]. As shown in Table 4, thermal conductivity increases with nanoparticle concentration. The thermal conductivity is nearly temperature independent for all studied nanofluids, and this temperature trend is quite similar to that of the base fluid. Thermal conductivity enhancements at 323.15 K are shown in Figure 7. Then, xGOnP and r-xGOnP have been compared with xGnP at this temperature, and, at the highest concentrations, the enhancement reaches 51.8% for xGnP/PG, 7.2% for xGOnP/PG, and 13.3% for r-xGOnP/PG. xGnP/PG nanofluids exhibit superior thermal conductivities than the others for the same weight fraction. 

These differences are because, after the oxidation part of the properties of the xGnP nanoparticles are lost, the thermal conductivity of xGOnP nanoparticles decreases drastically. After a reduction of xGOnP nanoparticles, part of these properties are recovered. Therefore, thermal conductivity of the r-xGOnP/PG nanofluids increases compared to xGOnP/PG nanofluids.

### 3.4. Non-Linear Viscoelastic Measurements

**Figure 8:**
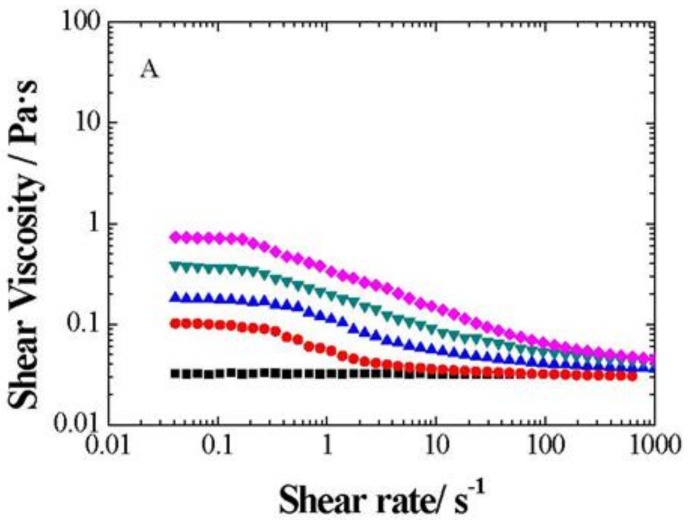

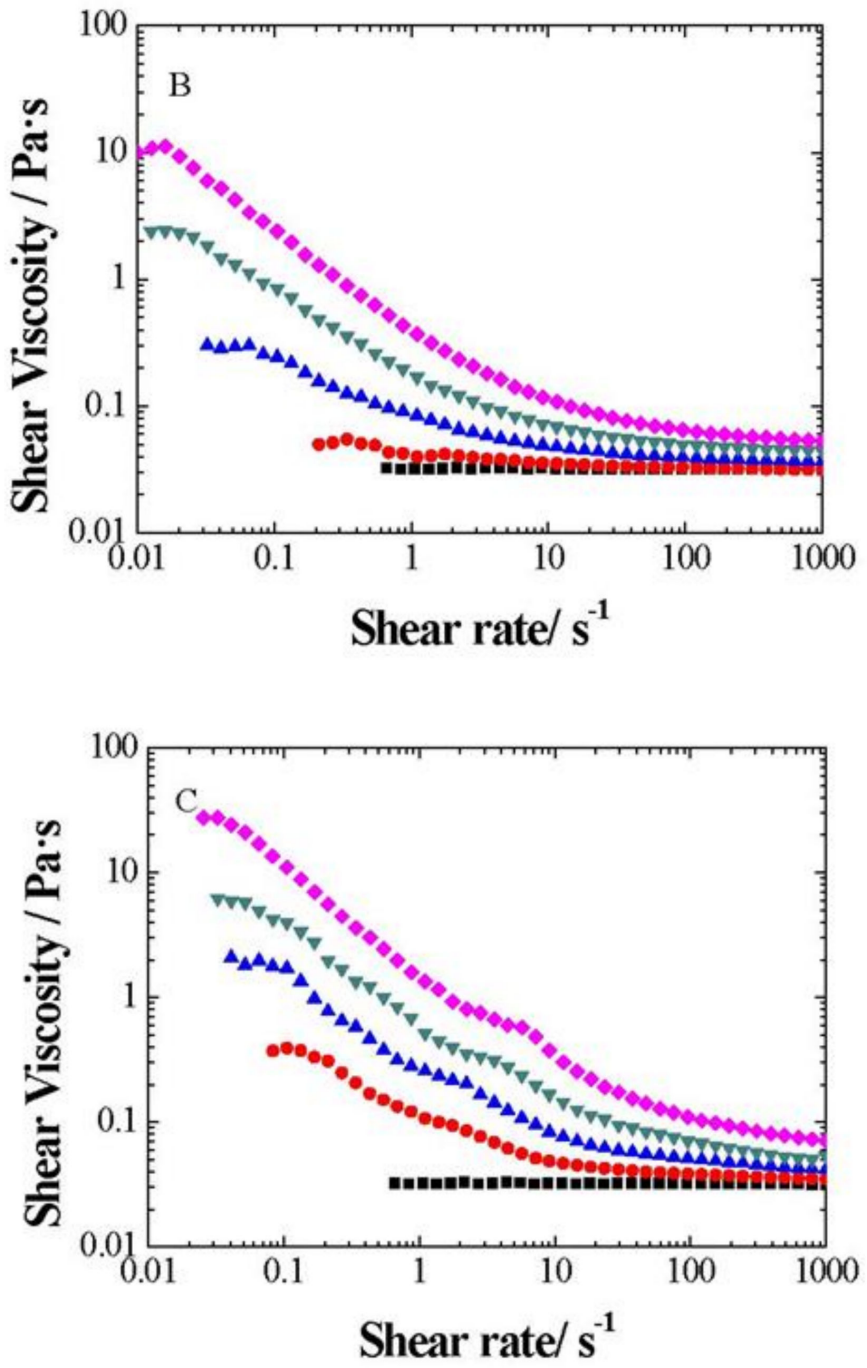
Shear viscosity change with shear rate (*γ*) for (**A**) xGnP/PG, (**B**) xGOnP/PG and (**C**) r-xGOnP/PG nanofluids, at 303.15 K for the concentrations: ■, PG; ● 0.5 wt. %, ▲ 1 wt. %, ▼2 wt. % and ♦ 3 wt. %.

Rheological data were measured starting with a shear viscosity variation with a shear rate. Figure 8 presents the flow curve, i.e., shear viscosity (μ) vs. shear rate (γ), for PG base fluid and four different weight fractions of xGnP/PG, xGOnP/PG, and r-xGOnP/PG nanofluids (0.5, 1, 2, and 3 wt %) at 303.15 K. The base fluid viscosity is Newtonian, that is, its value is shear rate independent. By opposition, all studied nanofluids present shear thinning behavior, which is a non-Newtonian behavior. The decreasing rate of shear viscosity is more prominent at a higher shear rate. A Newtonian plateau appears in the lowest shear rate region for more concentrated samples, and, in these cases, the stronger sheet-sheet and multi-sheet interactions produced a more pronounced shear thinning property effect. Shear thinning fluids are considered sensitive to the steepness of the viscosity flow curve, which provides a way to considerably decrease the flow resistance. This means that the shearing orients the particles in the direction of flow and its gradient. This can break agglomerates and, hence, reduce the amount of solvent immobilized by the particles. The interaction forces may then decrease and cause lowering in the flow resistance and the apparent viscosity of the system. Thus, the appearance of shear thinning in colloidal dispersions is clearly related to the modifications in the inter-particle structure [32], which provides a powerful tool to explore these internal structural rearrangements.

**Figure 9:**
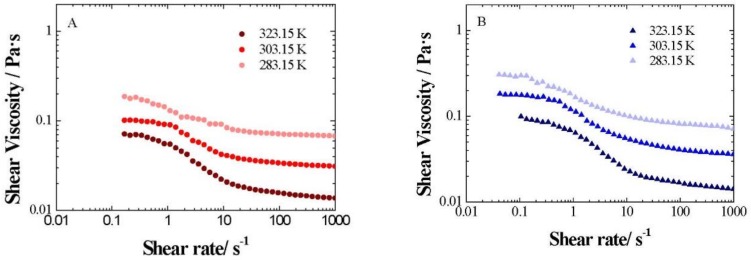

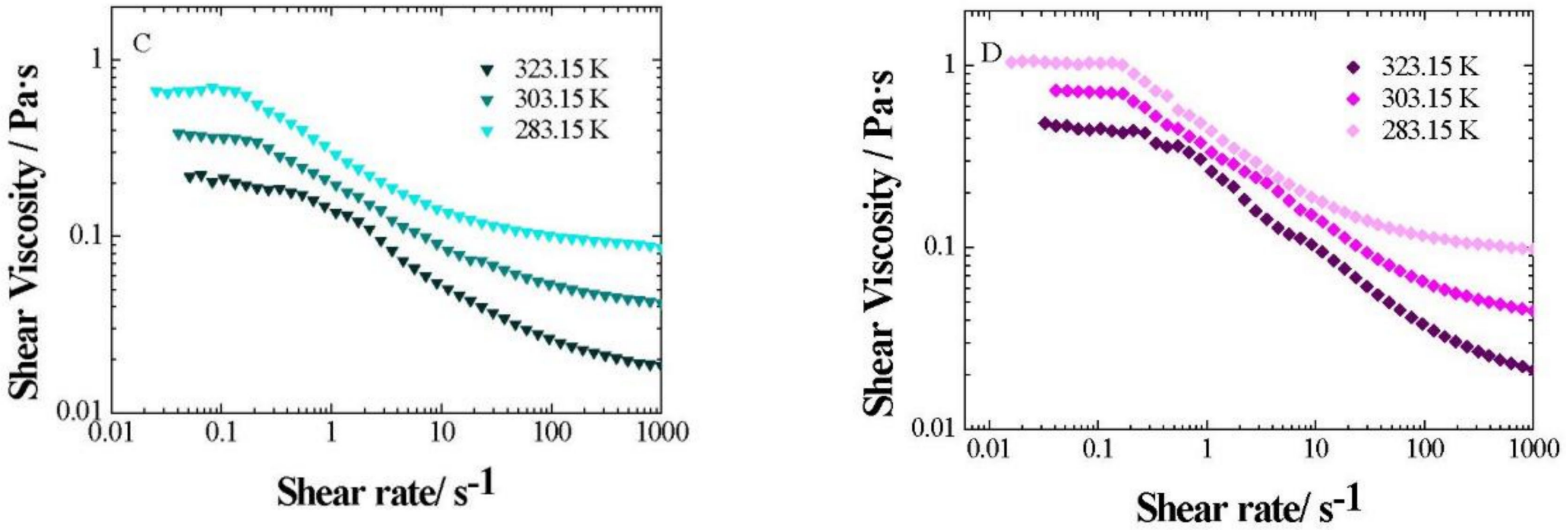
Shear viscosity change with shear rate (*γ*) for xGnP/PG nanofluids at different weight fractions: (**A**) 0.5 wt%, (**B**) 1 wt. %, (**C**) 2 wt. % and (**D**) 3 wt. %, between 283.15 and 323.15 K.

The influence of temperature on the flow curves was additionally studied for xGnP/PG nanofluids at different weight fractions between 283.15 and 323.15 K, as shown in Figure 9, where the expected viscosity decrease with an increasing temperature is observed.

## 4. Conclusions

Two different products have been synthesized from commercial graphene nanoplatelets (xGnP), with the aim to improve the nanoparticles stability in the base fluid. The characterization of the samples shows that the surface of graphene nanosheets has been oxidized successfully, and then completely reduced, which decreases the oxygen content in the surface of graphene and improves the nanofluid stability for both functionalized graphene nanofluids.

An experimental thermal conductivity characterization has been performed for xGnP/PG, xGOnP/PG, and r-xGOnP/PG nanofluids. Thermal conductivity increases with respect to the base fluids up to values of 51.8% for xGnP/PG, 7.2% for xGOnP/PG and 13.3% for r-xGOnP/PG, which reveals that, after oxidation, graphene oxide nanosheets lose part of their properties and produce a sharp decrease in the thermal conductivity of xGOnP/PG. After reduction, we recover part of these properties, which is evident, since the thermal conductivity of r-xGOnP/PG nanofluids increases. The influence of temperature in the thermal conductivity of xGnP/PG nanofluids shows that the thermal conductivity enhancements are almost temperature-independent.

Concerning rheological behavior, xGnP/PG, xGOnP/PG, and r-xGOnP/PG nanofluids show non-Newtonian behavior under the conditions implied in this work. As concentration increases, wider and clear Newtonian plateaus are found in the low shear rate region. This implies that, although the nanofluid thermal conductivity enhancement is positive in the potential framework of its application as heat exchange fluid, a careful evaluation of the microscopic flow conditions in every particular case is essential to determine if its viscosity will impose practical limitations for its use.

In conclusion, the chemical reduction process followed to obtain r-xGOnP improves the profile of the studied NF because it improves stability if compared with the original xGnP NF, which recovered an important part of the thermal conductivity that is lost during the previous surface oxidation procedure. Surface chemical functionalization appears then to be a useful design and tailoring tool that must be carefully evaluated since it offers different alternative routes to tune a given nanofluid thermo-physical profile.

## Figures and Tables

**Table 1:** XPS analysis results for xGnP, xGOnP, and r-xGOnP.

	Elemental Composition (% At)		Element Ratio
Sample	C	O	O/C
xGnP	95.23	3.92	0.041
xGOnP	69.16	29.47	0.426
r-xGOnP	90.52	8.91	0.098

**Table 2:** Interpretation of the de-convoluted C1s peaks and their relative atomic percentages, obtained from XPS results, for xGnP, GONs, GONs-OA, and GONs-OA-PP.

	Concentrations of C1s Functional Groups (%)				
	C1	C2	C3	C4	C5
Assignments	–C=C 285 eV	–C–C– 285.6–285.8 eV	–C–O–H, C–O–C 286.6–286.8 eV	O–C=O/C(O)=O 288–289.2 eV	π-π* 290.2 eV
xGnP	73.20	5.61	10.84	5.00	5.34
xGOnP	50.40	-	39.94	8.96	0.69
r-xGOnP	64.70	3.04	21.24	5.01	4.97

**Table 3:** Atomic percentage of the principal elements present in xGnP, xGOnP, and r-xGOnP, obtained by the analysis of the EDS spectra.

Element	xGnP	xGOnP	r-xGOnP
C (Atomic %)	95.36	68.85	90.57
O (Atomic %)	4.20	30.25	9.06

**Table 4:** Thermal conductivities of xGnP, xGOnP, and r-xGOnP/PG nanofluids at different weight fractions and atmospheric pressures.

T/K	k/W·m^−1^·K^−1^					
	0 wt. %	0.5 wt. %	1 wt. %	1.5 wt. %	2 wt. %	3 wt. %
	xGnP/PG					
283.15	0.196	0.214	0.226	0.245	0.259	0.297
303.15	0.195	0.212	0.224	0.246	0.261	0.298
323.15	0.195	0.212	0.228	0.248	0.263	0.296
	xGOnP/PG					
323.15	0.195	0.198	0.203	0.205	0.207	0.208
	r- xGOnP/PG					
323.15	0.195	0.203	0.210	0.213	0.216	0.221
